# Predictors of early mortality among patients with acute‐on‐chronic liver failure

**DOI:** 10.1002/jgh3.12557

**Published:** 2021-05-19

**Authors:** Bershic Valantine, Nabakishore Sundaray, Debakanta Mishra, Samir Sahu, Jimmy Narayan, Baikuntha N Panda, Ayaskanta Singh

**Affiliations:** ^1^ Department of General Medicine IMS and SUM Hospital, Siksha 'O' Anusandhan, Deemed to be University Bhubaneswar India; ^2^ Department of Gastroenterology IMS and SUM Hospital, Siksha 'O' Anusandhan, Deemed to be University Bhubaneswar India

**Keywords:** acute‐on‐chronic liver failure, bilirubin, model for end‐stage liver disease, mortality predictors, serum ferritin

## Abstract

**Background and Aim:**

Acute‐on‐chronic liver failure (ACLF) is a transpiring entity, which possesses high short‐term/early mortality (28 days). Several mortality predictors have been studied, but none were proved reliable. Serum ferritin, an acute phase reactant and marker of hepatic necro‐inflammation, is found to predict mortality in multiple liver diseases. We aimed to evaluate the role of serum ferritin and other clinical features, biochemical parameters and conventional scoring systems in predicting early mortality among ACLF.

**Methods:**

A prospective cohort study was done from October 2017 to March 2019 at a tertiary care (non‐transplant) center in eastern India. A total of consecutive 50 ACLF patients diagnosed, based on Asia Pacific Association for the Study of liver disease definition, were investigated for ferritin and other laboratory parameters on day‐0, day‐7, and followed up for 28 days.

**Results:**

Although the majority did not have organ failure (ACLF grade 0) according to European Association for Study of Liver‐chronic liver failure sequential organ failure assessment criteria, early mortality was high (56%). On undergoing univariate analysis, multiple variables (ascites, HE, creatinine, total leucocyte count (TLC), bilirubin, albumin) predicted mortality. However, on multivariate analysis, only total bilirubin independently predicted. None of the scores on day‐0 were predictive, while model for end‐stage liver disease [area under the receiver operating characteristics (AUROC)‐0.703, 95% confidence interval [CI]: 0.535–0.859] and Child–Turcotte–Pugh (AUROC‐0.697, 95% CI: 0.550–0.855) on day‐7 did.

**Conclusion:**

ACLF is a dynamic process; day‐7 assessment with above predictors, to be considered a milestone for prognostication and opting treatment modalities. Serum ferritin does not predict early mortality in ACLF.

## Graphical abstract

A prospective cohort was done in a tertiary non‐transplant center in eastern India, in order to evaluate the predictors of early mortality in acute‐on‐chronic liver failure. The well‐known inflammatory marker, serum ferritin was hypothesized as a novel mortality predictor and was also evaluated. The study revealed serum bilirubin, Child–Turcotte–Pugh score, and model for end‐stage liver disease, on day‐7 following admission, as the independent predictor of mortality; serum ferritin failed to predict.

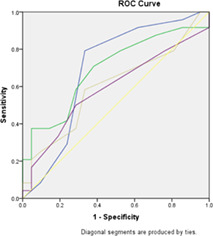

## Introduction

Acute‐on‐chronic liver failure (ACLF) is a transpiring entity characterized by acute deterioration of chronic liver disease. The deterioration is robust that causes multiple organ failure resulting in high short‐term (28 days) mortality, which ranges widely from 50–90%.^1^ Since no universal definition exists till date, consensus definition by Asia Pacific Association for the Study of liver disease (APASL) and European Association for Study of Liver‐American Association for Study of Liver Diseases (EASL‐AASLD) is well recognized.^2^, ^3^ Overall, ACLF pathogenesis is considered to be a “state of Immune dysfunction.”^4^, ^5^ Prognostication plays an inevitable role in clinical practice; by identifying patients with a probability of 28 days mortality, early management of the precipitating cause, prioritization for urgent liver transplantation, and feasibility of artificial liver support system can be planned and counseled. Multiple parameters and scoring systems such as Child–Turcotte–Pugh (CTP),^6^ model for end‐stage liver disease (MELD),^**7**^ Acute Physiology And Chronic Health Evaluation (APACHE),^8^ Sequential Organ Failure Assessment (SOFA)^9^ and chronic liver failure‐sequential organ failure assessment (CLIF‐SOFA) grading based on the EASL‐CLIF acute‐on‐chronic liver failure in cirrhosis (CANONIC) Study had been evaluated for the same. But none were conclusive, and discrepancies continued to remain. So a novel prognostic marker is the need of the hour.

Ferritin is a known acute phase reactant, which is also elevated in chronic inflammatory states.^10^ Hyperferritinemia and increased hepatic iron are found in chronic liver disease (CLD), specifically caused by hereditary Hemochromatosis, non‐alcoholic fatty liver disease, alcohol‐related and viral related chronic liver diseases.^11^ The pathophysiology is identified as impaired hepcidin production leading to disinhibited iron absorption and poor iron sensing of the liver cells. The cytosol protein ferritin is also released during any damage to hepatocytes and correlates with ALT levels. Hence it is considered as a surrogate marker of hepatic necro‐inflammation.^11^ Footing, in the recent past, multiple studies had identified ferritin as a predictor of mortality in decompensated cirrhosis.^11^, ^12^ Hence ACLF, which has CLD, inflammation, and hepatocellular damage, would be not an exception. This study was undertaken to evaluate the role of serum ferritin in predicting 28 days mortality and to identify other mortality predictors, so as to bring out the best short‐term mortality predictor of ACLF.

## Methods

This is a single‐centered prospective observational cohort study, undertaken in the Department of Internal Medicine and Department of Gastroenterology at Institute of Medical Science and SUM Hospital, Bhubaneswar; a tertiary care hospital (non‐transplant) in Eastern India. The study was conducted from October 2017 to March 2019. Consecutive ACLF patients, a total of 50, were included in the Study.

### Inclusion criteria

Patients diagnosed as ACLF based on APASL criteria within the age group of 18–79 years.

The APASL criteria of ACLF:

In a patient with recent or prior history of chronic liver disease (CLD), development of:

- Ascites on physical examination (and/or)
- Clinical encephalopathy

Within 4 weeks of the onset of:

- Jaundice (serum bilirubin ≥5 mg/dL) (and)
- Coagulopathy [International Normalized Ratio (INR) ≥1.5 or prothrombin activity <40%]

Infection is considered as acute precipitation, although APASL do not consider.

### Exclusion criteria

Patients fulfilling the APASL criteria found to have any of the following were excluded: previously known decompensated chronic liver disease (DCLD), patients who fail to follow up, iron deficiency anemia, patients following organ transplants (bone marrow), patients who fit the criteria for primary hemophagocytic lymphohistiocytic syndrome, primary iron and storage disorders like hemochromatosis. Conditions associated with secondary iron overload: thalassemia, congenital dyserythropoietic anemias, Friedreich's Ataxia, patients with chronic inflammatory diseases: rheumatoid arthritis, Systemic Lupus Erythematosus (SLE), and HIV.

### Methodology

The data about each patient were recorded in a predesigned format. A detailed history targeted to identify the varied modes of presentation, etiology of underlying CLD, and various acute insult was taken. Patients were examined thoroughly with an intent to determine the vitals, general status, severity of liver/organ failure, and stigma of underlying CLD. The patients were subjected to detailed laboratory investigations, which include complete blood count, serum electrolytes, viral serology, liver function tests, renal function test, prothrombin time, INR, urine routine microscopy, and serum ferritin. The examination and laboratory investigations were repeated on day‐7 and followed up totally for a duration of 28 days.

The diagnosis of CLD was based on standard laboratory and imaging techniques. In patients suspected of infection, investigations included chest X‐ray, echocardiography, and cultures from blood, urine, sputum, and ascetic fluid. In suspected cases, if two consecutive cultures fail to grow any, were considered as unknown infection. Evaluation was done for patients with unknown etiology of CLD. MedCalc for Windows, version 15.0 was used to calculate CTP, MELD, and SOFA scores. CLIF‐SOFA is calculated online at www.clifconsortium.com.

All the patients received treatment based on the institutional protocol for Cirrhosis, Complications of cirrhosis if present, and specific etiologies or acute precipitating event. Informed written consent was taken before the inclusion. Institutional Ethical Committee approval was obtained before the conduct of the study.

### Statistical analysis

The normally distributed quantitative variables were expressed as mean ± SD and categorical variables as frequency (percent). The non‐normal quantitative data were expressed as medians with interquartile range (IQR). The normally distributed quantitative and categorical variables were compared using Student's *t‐*test and Chi‐square test, respectively. The nonparametric unpaired data were compared using the Mann–Whitney *U* test. Univariate and multivariate logistic regression analysis with odds ratio was done to ascertain the predictors of mortality. A *P* value of <0.05 was considered statistically significant.

## Results

A total of 66 consecutive patients were recruited upon fulfillment of inclusion and exclusion criteria. All patients conveyed their willingness toward participation, except 5, who denied a written consent. The number of patients who lost to follow‐up was 11, including four who left against medical advice, making a cumulative of 50. Majority were males, 39 (78%), whereas only 11 (22%) were females. The mean age of presentation was 46.32 ± 12.72 years; amongst, the major half 22 (44%) fell within an age group of 36–55 years.

A detailed history and physical examination in most instances are sufficient enough to make a diagnosis of ACLF. The pattern of clinical presentation among our patients is as given by; infective symptoms (26%), bleeding manifestations (20%), icterus (100%), clinical ascites (84%), HE (30%), Splenomegaly (28%), Spider naevi (24%), and Caput medusa (10%).

### Chronic insult

The etiology of underlying CLD if found to be alcohol in 24 (48%), HBV related in 08 (16%), NASH in 3 (06%), and autoimmune hepatitis in 01 (02%). Etiology could not be detected among 14 (28%) after extensive workup and were considered cryptogenic. No significant statistical differences were found in the outcome of taking various etiologies (*P* value of 0.640).

### Acute insult

Infection was the predominant factor found to cause ACLF in previously stable CLD in our Study. Of the 22 (44%) patients who had infection, pneumonia was the main source in 6/22 (27%), followed by gastroenteritis 5/22 (23%), urinary tract infection 3/22 (14%), spontaneous bacterial peritonitis 2/22 (09%), and cutaneous infections 2/22 (09%); while foci/organism could not be identified in 4/22 (18%). The next leading causes were active alcohol intake 8 (16%) and drug‐induced liver injury 4 (8%). Hepatitis B reactivation was found among 03 (06%); a similar percentage was established to be Hepatitis B flare. Acute hepatitis‐A and Acute Hepatitis‐E were noticed among 2 (04%) and 1(02%), respectively. On the other hand, a significant proportion of the patients did not have any identifiable precipitating factors 14 (28%). However, the various acute insults noted did not have any influence on the outcome (*P* value of 0.254).

### Organ failure and mortality

On following up the study participants for 28 days, more than half (56%) died; of them, 4 (8%) expired within the first week. While assessing organ failure based on EASL criteria, liver (48%), coagulation (18%), renal (08%) were frequently affected on day‐0. Constantly the very same picture remained on day‐7 (liver [56%], coagulation [12%], renal [10%]), but it is noticeable that the other half did not have any sort of organ failure. Although APASL did not emphasize much on organ failures, explicating organ failures based on APASL; Liver failure was seen in all the patients on both days. Otherwise, renal failure was seen among 11 (22%), 6 (13%) on respective days; rest, as on the application of EASL. However, on screening for organ dysfunctions as per APASL, Cerebral dysfunction was noticed among 17 (34%) and 16 (35%) alongside renal dysfunction among 11 (22%) and 11 (25%) on respective days.

### Predictors of mortality

On univariate analysis, patients who presented with larger grades of ascites (*P* value of 0.041) and high serum creatinine (*P* value of 0.046) were found to have a high chance of mortality by day‐28, whereas the pre‐existing scores failed to show any (Table 1). While applying the same on day‐7, we observed patients who had clinically severe grades of ascites (*P* value of 0.002) and higher grades of hepatic encephalopathy (*P* value of 0.017) had high death rates; the same with higher levels of total leucocyte count (*P* value of 0.008), creatinine (*P* value of 0.022), bilirubin (*P* value of 0.040), and low albumin (*P* value of 0.049) (Table 2). In addition, higher MELD scores (*P* value of 0.018) and CTP scores (*P* value of 0.020) were also associated with mortality, when SOFA and CLIF‐SOFA did not. However, it is to be noticed that serum ferritin, the principal investigation molecule of our study, did not predict mortality on baseline and day‐7 (*P* values of 0.211 and 0.367, respectively).

**Table 1:** Baseline parameters between alive and dead on admission

Variables	Alive (*n* = 22)		Dead (*n* = 28)		Total (*n* = 50)		*P* value
Age ($\bar{x}$ (*σ*))	47.14	11.39	45.50	14.05	46.32	12.72	0.402
Sex (*n*,%)							
Male	19	86%	20	71%	39	78%	0.206
Female	03	14%	08	29%	11	22%	
Sepsis (*n*, %)							
Present	05	23%	08	28%	13	26%	0.197
Absent	17	77%	20	72%	37	74%	
GI bleed (*n*, %)							
Present	05	23%	05	18%	10	20%	0.669
Absent	17	77%	23	825	40	80%	
SIRS (*n*, %)							
Absent	01	05%	02	07%	03	06%	
Grade‐1	04	18%	06	22%	10	20%	0.838
Grade‐2	09	41%	11	39%	20	40%	
Grade‐3	06	27%	06	21%	12	24%	
Grade‐4	02	09%	03	11%	05	10%	
Ascites (*n*, %)							
Absent	06	27%	02	07%	08	16%	
Mild	03	14%	08	28.5%	11	22%	**0.041**
Moderate	09	41%	08	28.5%	17	34%	
Large	04	18%	10	64%	14	28%	
HE (*n*, %)							
Absent	17	77%	18	64%	35	70%	
Grade‐1	04	18%	05	18%	09	18%	0.240
Grade‐2	00	00%	04	14%	04	08%	
Grade‐3	01	05%	00	00%	01	02%	
Grade‐4	00	00%	01	04%	01	02%	
TLC ($\tilde{X},$ IQR)	12.65	8.90–14.50	12.60	10.14–18.50	12.65	9.27–25.19	0.384
TPC ($\tilde{X},$ IQR)	167	106–280	137	97.25–209.50	150.50	99.50–223.75	0.452
Creatinine ($\tilde{X},$ IQR)	0.85	0.60–1.07	1.02	0.80–1.67	0.95	0.70–1.42	**0.046**
Sodium ($\tilde{X},$ IQR)	130.50	124.25–134	130	126.25–134	130	126–134	0.702
Bilirubin ($\tilde{X},$ IQR)	9.90	7.47–17.11	12.95	8.92–12.95	11.77	8.37–17.20	0.278
ALT ($\tilde{X},$ IQR)	47	35.50–187.25	79	47.50–167.25	61	40–164	0.269
Albumin ($\tilde{X},$ IQR)	2.50	2.29–3.40	2.50	2.02–2.90	2.50	2.20–3.00	0.272
INR ($\tilde{X},$ IQR)	1.85	1.50–2.45	1.80	1.60–2.23	1.80	1.57–2.32	0.806
Ferritin ($\tilde{X},$ IQR)	760.50	244.25–1240.25	853.50	462.50–1843.50	853	352–1410	0.211
MELD ($\bar{x}$, *σ*)	24.18	3.93	25.68	5.29	24.93	4.61	0.274
SOFA ($\tilde{X},$ IQR)	4.50	3.00–6.25	05	4.00–6.00	05	3.75–5.00	0.197
CTP (*n*, %)							
Class‐A	00	00%	00	00%	00	00%	
Class‐B	04	18%	04	14%	08	16%	0.392
Class‐C	18	82%	24	86%	42	84%	
ACLF (*n*, %)							
Grade‐0	19	86%	18	63%	37	74%	0.626
Grade‐1	00	00%	04	14%	04	08%	
Grade‐2	02	09%	04	14%	06	12%	
Grade‐3	01	05%	02	09%	03	06%	

$\tilde{X\;}$, median, *n*, count, $\bar{x}$, mean, *σ*, SD.

ACLF, acute‐on‐chronic liver failure; ALT, alanine aminotransferase; CTP, Child‐Turcotte‐Pugh; IQR, interquartile range; MELD, model for end‐stage liver disease; SIRS, systemic inflammatory response syndrome; SOFA, sequential organ failure assessment; TPC, total platelet count.

**Table 2:** Parameters between alive and dead on day‐7

Variables	Survivors (*n* = 22)		Dead (*n* = 24)		Total (*n* = 46)		*P* value
Sepsis (*n*, %)							
Present	04	18%	05	21%	09	20%	0.821
Absent	18	82%	19	79%	37	80%	
GI bleed (*n*, %)							
Present	01	05%	05	21%	6	13%	0.085
Absent	21	95%	19	79%	40	87%	
SIRS (*n*, %)							
Absent	05	23%	04	16%	09	20%	0.161
Grade‐1	10	45%	07	29%	17	47%	
Grade‐2	05	23%	07	29%	12	26%	
Grade‐3	01	4.5%	05	21%	06	13%	
Grade‐4	01	4.5%	01	04%	02	04%	
Ascites (*n*, %)							
Absent	05	23%	02	08%	07	35%	**0.002**
Mild	04	18%	06	25%	10	22%	
Moderate	09	41%	03	13%	12	26%	
Large	04	18%	13	54%	17	37%	
HE (*n*, %)							
Absent	19	86%	10	42%	29	41%	**0.017**
Grade‐1	02	09%	06	25%	08	17%	
Grade‐2	01	05%	07	29%	08	17%	
Grade‐3	00	00%	01	04%	01	05%	
Grade‐4	00	00%	00	00%	01	02%	
TLC ($\tilde{X},$ IQR)	10.90	7.30–14.02	14.15	11.10–21.17	12.26	9.47–17.80	**0.008**
TPC ($\tilde{X},$ IQR)	150	108–251	145	100–193	150	100–215	0.567
Creatinine ($\tilde{X},$ IQR)	0.80	0.67–0.90	1.00	0.72–1.40	0.90	0.70–1.00	**0.022**
Sodium ($\tilde{X},$ IQR)	132	128.75–134.75	131	129–134	132	129–134	0.635
Bilirubin ($\tilde{X},$ IQR)	10.45	7.77–17.15	17.40	8.55–23.57	13.30	8.12–20.50	**0.040**
ALT ($\tilde{X},$ IQR)	47.5	34.50–183.50	57.50	34.25–123.25	56	35–155	0.792
Albumin ($\tilde{X},$ IQR)	2.60	2.37–3.14	2.50	2.12–2.60	2.53	2.20–2.80	**0.049**
INR ($\tilde{X},$ IQR)	1.55	1.47–2.20	1.80	1.65–2.20	1.80	1.57–2.20	0.096
Ferritin ($\tilde{X},$ IQR)	952.50	296–1499.75	1087.50	445.50–1848.25	996	375.25–1769.50	0.367
MELD ($\tilde{X},$ IQR)	22.50	20.00–25.25	25.00	23.00–29.75	24.00	22–27	**0.018**
∆MELD ($\tilde{X},$ IQR)	−1.00	−3.00‐1.25	1.00	−1.75‐4.00	0.00	−2.25‐3.00	0.647
SOFA ($\tilde{X},$ IQR)	04	4.00–6.00	04	4.00–6.00	04	4.00–6.00	0.214
CTP (*n*, %)							
Class‐A	00	00%	00	00%	00	00%	**0.020**
Class‐B	08	36%	2	08%	10	22%	
Class‐C	14	64%	22	92%	36	78%	
ACLF (*n*, %)							
Grade‐0	19	86%	16	67%	35	76%	0.295
Grade‐1	01	05%	3	13%	04	09%	
Grade‐2	01	4.5%	4	16%	05	11%	
Grade‐3	01	4.5%	1	04%	02	04%	

$\tilde{X\;}$, median; *n*, count, $\bar{x}$, mean, *σ*, SD.

ACLF, acute‐on‐chronic liver failure; ALT, alanine aminotransferase; CTP, Child‐Turcotte‐Pugh; IQR, interquartile range; MELD, model for end‐stage liver disease; SIRS, systemic inflammatory response syndrome; SOFA, sequential organ failure assessment; TPC, total platelet count.

**Figure 1:**
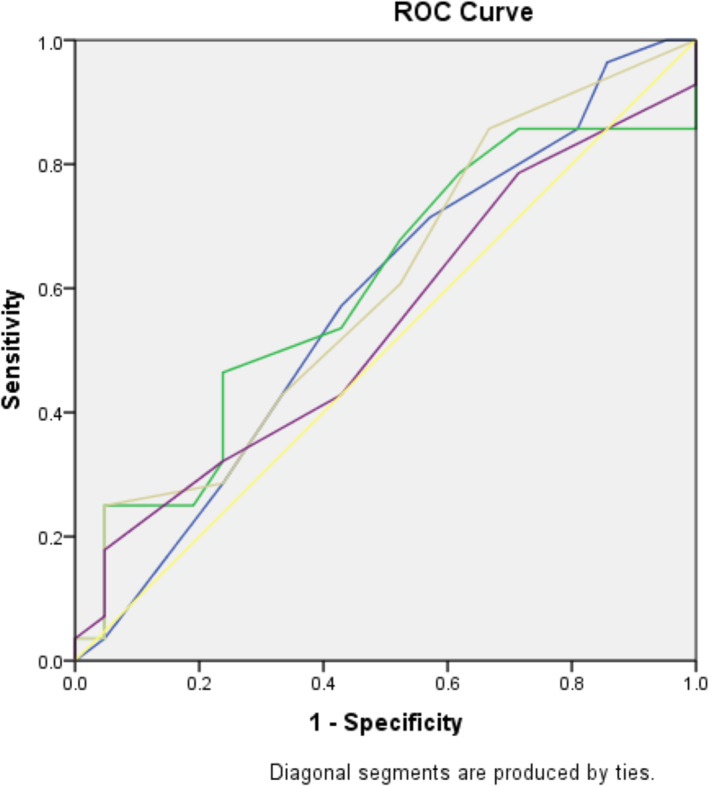
Receiver Operating Characteristic (ROC) curves of scores predicting mortality on day‐0. CLIF, chronic liver failure; CTP, Child‐Turcotte‐Pugh; MELD, model for end‐stage liver disease; SOFA, sequential organ failure assessment. Source of the curve: 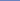, CTP0; 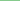, MELD0; 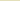, SOFA0; 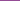, CLIF0; 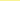, reference line.

On execution of binary logistic regression, serum Bilirubin on day‐7 (*P* = 0.048) is found to be an independent predictor of mortality (Table 3). To explore the accuracy of the day‐7 scores in predicting mortality, a receiver operating curve (ROC) plot was performed (Fig. 1, Table 4), but none was found superior (MELD AUROC‐0.703, CTP AUROC‐0.697) (Table 5).

**Table 3:** Predictors of mortality in univariate and multivariate analyses

Parameters	Univariate analysis			Multivariate analysis		
	Odds ratio	95% Confidence interval	*P* value	Odds ratio	95% Confidence interval	*P* value
Day‐0						
Creatinine	1.916	1.06–5.404	0.04	1.55	0.89–2.73	0.371
Day‐7						
HE						
Grade‐1	5.7	0.967–33.6	0.054	2.284	0.286–18.247	0.436
Grade‐2	13.3	1.429–123.79	0.023	6.725	0.426–106.233	0.176
Grade‐3	3.07	1.12–5.21	0.03	7.32	0.58–10.23	0.340
TLC	1.136	1.008–1.28	0.03	1.012	0.885–1.156	0.867
Creatinine	2.62	1.18–8.51	0.04	1.232	0.32–4.738	0.761
Bilirubin	1.11	1.01–1.22	0.03	1.151	1.001–1.323	**0.048**
Albumin	0.171	0.036–0.81	0.02	0.136	0.015–1.215	0.074

Factors adjusted in the multivariate analysis for day‐0 predictors of outcome are age and sex.

**Table 4:** Results from the area under ROC, for scores predicting mortality on day‐0

Score	Area	Standard error	*P* value	95% Confidence interval		Cutoff value	Sensitivity	Specificity
				Lower bound	Upper bound			
CTP	0.577	0.084	0.363	0.411	0.742	11	71.40%	42.90%
MELD	0.597	0.082	0.249	0.436	0.758	24	67.90%	47.60%
SOFA	0.599	0.083	0.237	0.438	0.761	05	60.70%	47.60%
CLIF	0.54	0.083	0.635	0.377	0.703	09	42.90%	57.10%

CLIF, chronic liver failure; CTP, Child‐Turcotte‐Pugh; MELD, model for end‐stage liver disease; SOFA, sequential organ failure assessment.

**Table 5:** Results from the area under ROC, for scores predicting mortality on day‐7

				95% Confidence interval				
Score	Area	Standard error	*P* value	Lower bound	Upper bound	Cutoff value	Sensitivity	Specificity
CTP	0.697	0.083	0.022	0.535	0.859	12	79.20%	68.20%
MELD	0.703	0.078	0.019	0.55	0.855	24	70.80%	63.60%
SOFA	0.6	0.085	0.251	0.433	0.767	05	58.30%	66.70%
CLIF	0.588	0.086	0.311	0.42	0.757	09	50.00%	71.40%

CLIF, chronic liver failure; CTP, Child‐Turcotte‐Pugh; MELD, model for end‐stage liver disease; SOFA, sequential organ failure assessment.

## Discussion

ACLF is a transpiring entity, gaining the attention of physicians gradually. Around the country and the globe, limited studies have concentrated on the short‐term predictors of mortality; of them, only a minor are prospective studies. Among the total 50 ACLF patients, most (78%) were male, and the mean age of presentation was 46.22 ± 12.72 years. These exactly match the very picture of previous Indian studies.^13^, ^14^ The first component of any definition toward ACLF is the existence of underlying CLD. Alcoholic cirrhosis is the most among all the causes of CLD in the West,^2^, ^3^ whereas HBV related CLD in Asian countries. This trend had changed over the years when the recent Asian studies pointed to alcoholic cirrhosis instead;^13^, ^14^, ^15^ probably indicating westernization. Our study too evidences the same (48%). The next leading cause of CLD in our study was cryptogenic (28%), which might also be a presentation of NASH, as the proportion of obesity and NAFLD are on a rising trend.^16^, ^17^

ACLF is a distinct entity that is potentially reversible to the baseline clinical status if the acute/precipitating events recover.^18^, ^19^ As demonstrated in Western studies,^20^, ^21^ bacterial infections (44%) is identified as an important cause of decompensation among our patients. While studies from India and eastern countries have mentioned Hepatitis B infection as the common acute insult, our observation, though contradicting, could be explained by the emergence of awareness and advanced treatment protocols for Hepatitis‐B in recent years. Strengthening our observation, recent studies by Indian National Association for Study of the Liver (INASL), Kulkarani et al., Sharma et al., and APASL ACLF research consortium (AARC) have also shown the same.^14,15,21,22^ The next leading acute precipitant in our Study is active alcohol intake (16%), while the other precipitants were meager. It is intriguing that 14 (28%) did not have any identifiable acute precipitants. The aforementioned might have resulted from bacterial or fungal infections that have gone undetected and unrecognized drug‐induced liver injury.^23^ The acute precipitating events stated above match the very picture of CANONIC Study, Selva Rajoo et al. and Duseja et al.^3^, ^23^, ^24^ Irrespective of etiology of insults, acute severe liver dysfunction leads to the end result; severity decides the outcome.

The acute deterioration of liver function in ACLF leads to multi‐organ failure that results in high short‐term mortality. The short‐term mortality in this study is 56%. Similar results have been shown by Indian authors, Duseja et al., Jha et al., and Garg et al.^24^, ^25^, ^26^ and international bodies like EASL and APASL.^2^, ^3^ Over the last few years, multiple studies have worked on identifying the predictors of mortality,^27^ but most of them were retrospective studies and results were manifold. We, in an effort to identify the same, on the multivariate analysis found bilirubin as an independent predictor of mortality (Table 3). The serum bilirubin serves as an integral component in every score/definition/criteria towards ACLF. On a literature search, Yang SS demonstrated that higher bilirubin levels have a sensitivity of 94.1% and specificity of 87.5% among chronic hepatitis B ACLF.^28^ Ming‐Hua Zheng et al., in their study among chronic hepatitis B ACLF, have shown that bilirubin is independently associated with mortality both in long‐ and short‐term survival.^29^ López‐Velázque et al., in his cohort among 65 patients, had shown bilirubin as a sole mortality predictor when MELD and CTP failed.^30^ As a contribution, the RELIEF trial also has found bilirubin predicting mortality within 4 days.^31^ The conjugation ability of the Liver is impaired in ACLF, leading to unconjugated hyperbilirubinemia; on the other hand, Cholestasis in ACLF impairs the excretion of conjugated bilirubin, resulting in conjugated hyperbilirubinemia. In addition, deterioration of liver function caused by accumulated products of cholestasis in ACLF is rapid as compared to other cholestasis diseases; altered regulation of the farnesoid X receptor, androstane receptor, and pregnane X receptor, which are involved in the detoxification and transport of bile acids and bilirubin are identified to be the reason behind.^32^ Our study proposes total serum bilirubin on day‐7, a valuable tool in predicting the outcome.

**Figure 2:**
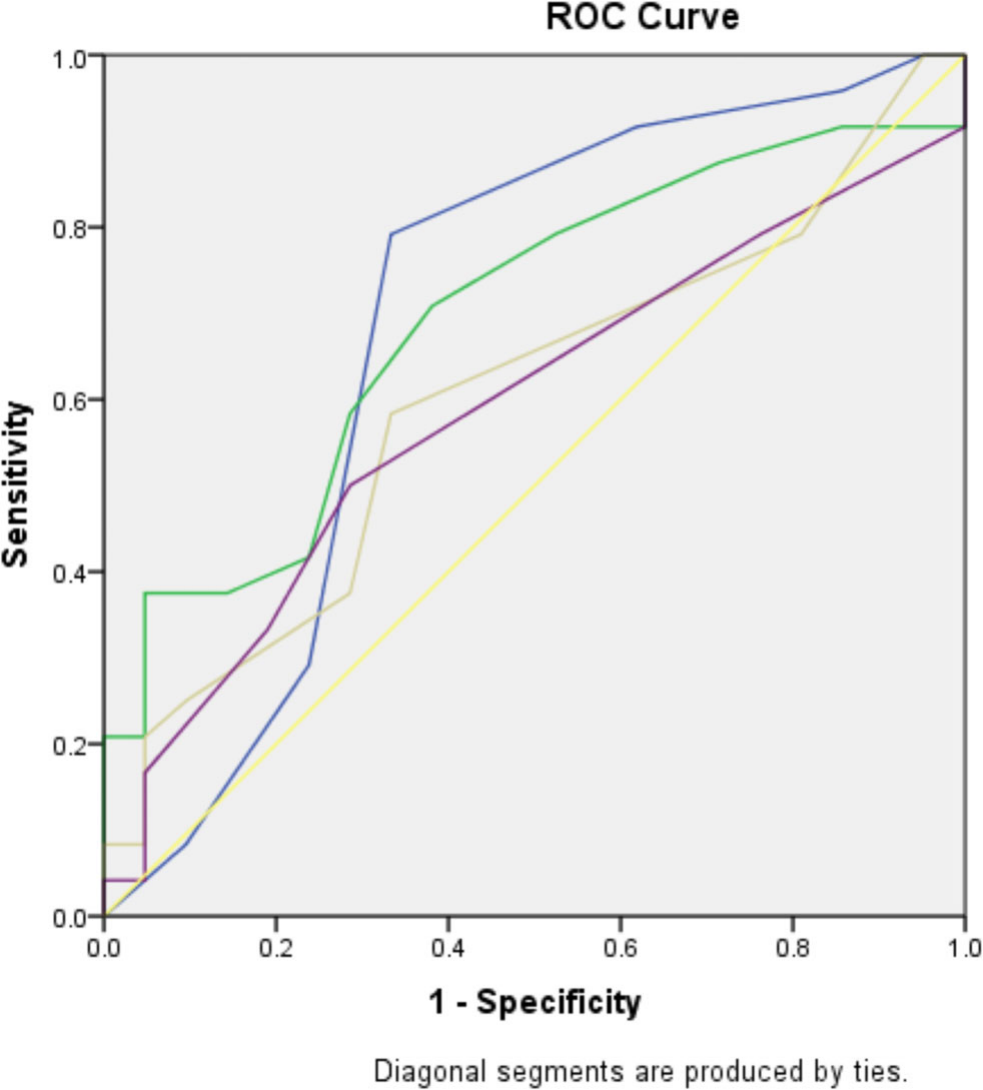
ROC curves of scores predicting mortality on day‐7. CLIF, chronic liver failure; CTP, Child‐Turcotte‐Pugh; MELD, model for end‐stage liver disease; SOFA, sequential organ failure assessment. Source of the curve: 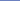, CTP7; 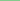, MELD7; 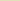, SOFA7; 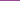, CLIF7; 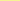, reference line.

On analysis, in order to validate conventional scores—MELD, CTP, SOFA, and newer score—CLIF‐SOFA in mortality prediction, none on day‐0 were predictive (Table 1), while MELD and CTP of day‐7 did (Table 2). Based on the comparison, MELD stands a minuscule taller (AUROC—0.703 ± 0.078, *P* < 0.019) than CTP (AUROC—0.697 ± 0.083, *P* < 0.022) (Fig. 1, Table 4). Similar results have been demonstrated by previous studies where MELD is observed superior to CTP and SOFA.^21^, ^26^, ^33^, ^34^ Flabbergasting, the CLIF‐SOFA, which is considered superior to the above ones in predicting mortality,^34^, ^35^ stays way behind in this study. The Indian authors Sharma et al., Kumar et al., and Duseja et al. have also brought out similar observations in their respective studies.^21^, ^33^, ^36^ MELD and CTP determine the severity of the underlying liver disease, whereas SOFA and CLIF‐SOFA assess the extent of extra‐hepatic organ involvement. It is quite obvious that organ failure resulting from any disease correlates proportionately with mortality; ACLF, acute severe decompensation of the liver, also results in organ failure and subsequent death. Accordingly, the organ failure scores are reflective of mortality than being predictive (Fig. 2).

When ACLF is a result of acute liver failure in CLD, the liver function targeted scores like MELD and CTP can identify their severity. But earlier studies have found, although having the same MELD score, mortality is more with ACLF when compared with AD.^37^ So the modification of the MELD score or consideration of a lower score may suffice to predict mortality among ACLF. Thus, none of the scores predict mortality effectively, and the vacuum remains. The baseline disease severity scores, that is, MELD, SOFA, etc. are not a predictor of outcome. This is probably because ACLF per se is an entity with high mortality (50–90%), which may be independent of severity scores. However, the improvement or deterioration of the patients' liver status, as reflected by liver severity scores (CTP, MELD) in the first week of disease course predicts the outcome.

A study done in Delhi by Maiwall et al. showed that serum ferritin, the chief element of this study, as an independent predictor of mortality.^11^ The same study has identified ferritin as a mortality predictor equivalent to CLIF‐SOFA grades and superior to MELD and CTP. Howbeit in this current study, although elevated 4–5 times from the baseline, no association prevails between the survivors and non‐survivors (*P* < 0.211, *P* < 0.367). Maras et al., in their study, to find the predictors of mortality among ACLF, evaluated iron regulating proteins and found that ferritin was significantly elevated among ACLF than CLD but did not show much variation between alive and dead (*P* = 0.63).^38^ A similar study by Burns et al. also exhibited that no correlation exists between survivors and dead with ferritin, although it is found elevated among both groups (*P* = 0.06).^39^ This could be explained by the fact that ferritin is a nonspecific acute phase reactant and may depend on an individual's iron stores. Although being a marker of hepatic necro‐inflammation, not more potent enough to predict mortality, as the reason for hyperferritinemia is multidimensional.

### Limitations

However, a larger sample size would have given a much clearer picture. This study also failed to analyze the association of other inflammatory markers in ACLF and other relevant prognostic scoring systems like APACHE II and AARC.

## Conclusion

Although not at the time of diagnosis, serum bilirubin, CTP, and MELD predicted mortality independently on day‐7. Serum ferritin does not predict early mortality in ACLF. ACLF is a dynamic process; improvement or deterioration of the patients' liver status, as denoted by liver severity scores (CTP, MELD), in the first week (hours of decision) of disease course predicts the outcome. Hence, day‐7 assessment using the above is to be considered a milestone for prognostication and opting treatment modalities.

### Summary

Single centered, prospective observational cohort study was carried out in a tertiary care hospital (non‐transplant) in eastern India, among 50 ACLF patients diagnosed based on APASL definition, to evaluate serum ferritin and other predictors of 28 days mortality. Alcohol was the most common cause of CLD. Bacterial infection is the predominant acute insult that led the stable CLD to ACLF. Mortality was 56%, even though the majority did not have organ failures. Neither parameters nor severity scores predicted mortality on admission; while, serum bilirubin, CTP, and MELD on day‐7 independently did. Serum ferritin was not associated with mortality in any of the days.
